# Evaluating the Sublethal Effects of *Origanum vulgare* Essential Oil and Carvacrol on the Biological Characteristics of *Culex pipiens* biotype *molestus* (Diptera: Culicidae)

**DOI:** 10.3390/insects14040400

**Published:** 2023-04-20

**Authors:** Athanasios Giatropoulos, George Koliopoulos, Pavlos-Nektarios Pantelakis, Dimitrios Papachristos, Antonios Michaelakis

**Affiliations:** 1 Laboratory of Efficacy Control of Pesticides, Benaki Phytopathological Institute, 14561 Kifissia, Greece; 2 Laboratory of Agricultural Zoology and Entomology, Agricultural University of Athens, 11855 Athens, Greece; 3 Laboratory of Agricultural Entomology, Benaki Phytopathological Institute, 14561 Kifissia, Greece; 4 Laboratory of Insects and Parasites of Medical Importance, Benaki Phytopathological Institute, 14561 Kifissia, Greece

**Keywords:** larvicides, sublethal effects, biology, growth inhibition, *Culex pipiens* biotype *molestus*, carvacrol, oregano oil

## Abstract

**Simple Summary:**

In recent decades, essential oils from various plant species have been extensively tested as low-risk larvicides showing acute toxicity and/or insect growth regulatory properties through various mechanisms of action on mosquitoes. In the laboratory, we evaluated the chronic effects of larvicidal sublethal (LC_50_) concentrations of essential oil from *Origanum vulgare* and its major component carvacrol on biological parameters of the principal West Nile virus vector *Cx. pipiens* biotype *molestus.* The short-term (24 h) treatment of mosquito larvae with LC_50_ concentrations produced significantly delayed mortality and morphological abnormalities to surviving larvae and pupae and resulted in failed adult emergence, indicating a potential growth inhibition mode of action for the tested materials. The results reported herein promote carvacrol and carvacrol-rich oregano oil as effective larvicides against *Cx. pipiens* biotype *molestus* at doses lower than the acute toxic ones, suggesting a use for these botanical insecticides that is safer for the environment and less costly.

**Abstract:**

*Culex pipiens* is a mosquito species complex spread worldwide that poses a serious threat to human health as the primary vector of West Nile virus. Its control is mainly based on larvicidal applications with synthetic insecticides on mosquito breeding sites. However, the excessive use of synthetic larvicides may provoke mosquito resistance issues and negative side effects to the aquatic environment and human health. Plant-derived essential oils, including those from the Lamiaceae family, can be eco-friendly alternative larvicidal agents causing acute larval toxicity and/or growth inhibitory effects on the developmental stages of mosquitoes through different modes of action. In the current laboratory study, we evaluated the sublethal effects of carvacrol-rich oregano essential oil and pure carvacrol on *Cx. pipiens* biotype *molestus*, the autogenous member of the *Cx. pipiens* species complex, after the exposure of 3rd–4th instar larvae to LC_50_ concentrations. The short-term (24 h) larvicidal treatment with the sublethal concentrations of both tested materials exhibited an acute lethal effect on the exposed larvae as well as significant delayed mortality for surviving larvae and pupae. Larvicidal treatment with carvacrol reduced the longevity of the emerged males. In addition, the morphological abnormalities that were observed at the larval and pupal stage along with failed adult emergence indicate the potential growth inhibitory properties of the tested bioinsecticides. Our findings suggest that carvacrol and carvacrol-rich oregano oil are effective plant-based larvicides at doses lower than the acute lethal ones, thus promoting an environmentally friendly and more affordable perspective for their use against the WNV vector *Cx. pipiens* biotype *molestus.*

## 1. Introduction

Mosquitoes are well-known vectors of several life-threatening diseases for humans such as malaria, dengue, and West Nile virus (WNV) [1]. WNV is considered the most widespread arbovirus in the world, with increasing geographic range and frequency of symptomatic infections in humans and animals over recent decades [2,3]. In Europe, since the first large-scale outbreak of WNV in 1996 in Romania, human cases of the disease have increased and are mainly spread in southern and southeastern European countries [4,5]. *Culex pipiens* (Linnaeus 1758), the common house mosquito, is a species complex native to Europe, where it acts as the principal vector in outbreaks of WNV infections [3,6]. Within the complex, *Cx. pipiens* biotype *molestus* is an autogenous mosquito (i.e., no blood meal required for first egg batch) feeding predominantly on mammals, including humans, with the ability to transmit WNV [7,8,9,10].

In the context of the integrated vector management (IVM) strategy for outbreak prevention of WNV infection in Europe, larval control plays a key role targeting immature stages of mosquitoes to keep *Cx. pipiens* females at density levels below the threshold that poses a public health risk [6]. Mosquito control management programs in Europe rely on the use of a limited number of microbial or synthetic larvicides approved as active substances under the EU regulation 528/2012, namely the microbial insecticides *Bacillus thuringiensis* subsp. *israelensis* and *Bacillus sphaericus*, the juvenile hormone mimics *s*-methoprene and pyriproxyfen, and the chitin synthesis inhibitor diflubenzuron [11]. However, the overuse of synthetic pesticides may lead to mosquito resistance issues [12,13] such as the recent high-level resistance cases of *Cx. pipiens* to diflubenzuron with focal distribution in Northern Italy [14,15]. Hence, there is great interest in identifying new efficient agents against mosquito larvae of the WNV vector *Cx. pipiens* with a safe profile for both the environment and humans.

Over the past two decades, plant-derived essential oils (EOs) and their major constituents, primarily terpenes, phenolics, and alkaloids, have been tested against mosquitoes as low-risk alternatives to synthetic insecticides [16,17,18]. In this respect, EOs as complex mixtures of many bioactive compounds act through multiple mechanisms of action on insects, so they are less prone to inducing resistance as compared to synthetic insecticides [19,20]. EOs from various plant species and their compounds exert larvicidal activities on different mosquito species [21,22,23,24,25] as neurotoxic agents that target acetylcholinesterase and GABA and octopamine synapses, insect growth regulators that disrupt the normal process of morphogenesis as well as reproductive inhibitors [20,26,27].

In the context of EOs, Lamiaceae is the most frequently cited plant family and *Origanum* is one of the most-reported genera within this family with significant insecticidal properties [17]. The monoterpene carvacrol and carvacrol-containing EOs from Lamiaceae aromatic plant species including *Origanum vulgare* have been reported for their toxic properties against mosquito larvae of the *Cx. pipiens* species complex [28,29,30,31,32,33,34]. The neurotoxic action of carvacrol on insects has been associated with acetylcholinesterase inhibition and octopamine and GABA receptors [35,36,37,38]. In field studies in Italy, both emulsified and crude carvacrol-rich essential oil from *Origanum vulgare* applied in road drains exerted high efficacy in terms of immature mosquito population reduction of *Cx. pipiens* and *Aedes albopictus* for 1–3 weeks [39,40].

Although EOs are promising plant secondary metabolites for the development of botanical mosquito larvicides, no commercially manufactured botanical products based on EOs are available in the European market [11,41], due to the following main reasons: (i) EOs are relatively expensive active substances because of the high plant yield sources usually required for their isolation, (ii) their chemical composition varies, which may result in varying biological activities, and (iii) they are quickly degraded or evaporated, which may reduce their efficacy [23]. However, these barriers may be overcome by selecting suitable chemotypes, using appropriate formulations, and optimizing some subsidiary properties of EOs. For example, the application of sublethal doses of some EOs may cause a significant decrease in the survival, fecundity, fertility, and longevity of insects, and, therefore, the lower-cost applications of lower doses may ultimately reduce population densities [23,42,43]. Due to the potential chemical instability of botanical larvicides when exposed to light and heat, it is important to explore their residual effect in laboratory and field trials as a key parameter for their efficacy evaluation.

In the laboratory, we previously tested the LC_50_ concentrations of carvacrol-rich oregano EO and carvacrol against *Ae. albopictus* larvae and we found significant inhibition of adult emergence and physiological abnormalities, indicating potential growth inhibitory activity of the tested materials [44]. As a follow-up, in the current study, we evaluated the effects of LC_50_ concentrations of the essential oil from *Origanum vulgare* (Lamiaceae), and its major component carvacrol, on the biological parameters of the WNV vector *Cx. pipiens* biotype *molestus*, namely larval and pupal survival and longevity, sex ratio of surviving adults, longevity of males and females, pre-oviposition period, fecundity, fertility, and adults’ body size.

## 2. Materials and Methods

### 2.1. Chemicals Tested

Oregano EO, dominated by carvacrol (69.8%), was extracted from plants of *Origanum vulgare* subsp. *hirtum* (Lamiaceae) that originated from Tirnavos, Larissa, Greece, and was supplied by Tharros SA, Tzioumakis Bros [44]. Carvacrol with high purity (99%) was supplied by Sigma-Aldrich (Steinheim, Germany).

### 2.2. Mosquito Colony

The mosquito larvae used in this study originated from a *Cx. pipiens* biotype *molestus* laboratory colony that was kept at T = 26–27 °C, R.H. = 50–60%, and photoperiod (L:D) = 16:8 h. Adult mixed-sex mosquitoes were maintained in cages (length × width × height: 33 cm × 33 cm × 33 cm) covered by mesh and were supplied with 10% sucrose solution. Females were not provided with blood for egg development due to autogeny. Larvae were reared in containers with tap water and fed ad libitum with dried wheat bread until pupation. Beakers with 100 mL water were placed into the rearing cages for egg laying.

### 2.3. Dose–response Bioassays (LC_50_ Determination)

Acute toxicity bioassays on mosquito larvae were performed following World Health Organization (WHO) guidelines [45] with slight modifications, as previously described by Giatropoulos et al. [44]. Briefly, oregano oil and carvacrol were dissolved in dimethyl sulfoxide (DMSO), preparing stock solutions of each test substance at a concentration of 10% *v*/*v*. Twenty late 3rd to early 4th instar larvae were placed into 100 mL water solution containing 2% *v*/*v* DMSO. Each tested substance solution was added into the water solution of 2% *v*/*v* DMSO, and then gentle shaking followed to create a homogeneous final test solution. Four replicates per dose (5, 10, 15, 20, 25, and 35 μL L^−1^ for oregano oil and 3, 5, 7, 8, 9, 11, and 13 μL L^−1^ for carvacrol) were employed, and a treatment with 100 mL water solution containing 2% *v*/*v* DMSO was included in each bioassay as a control. Larval mortality was recorded after 24 h exposure, and LC_50_ doses were determined.

### 2.4. Sublethal Effects after Exposure to Larvicidal LC_50_ Concentrations

#### 2.4.1. Survival and Longevity of Larvae and Pupae

Batches of 20 late 3rd to early 4th instar mosquito larvae were exposed for 24 h to previously calculated LC_50_ concentrations of oregano oil (19.9 μL L^−1^) and carvacrol (6.4 μL L^−1^) in 100 mL water solution of 2% *v*/*v* DMSO, and to 100 mL water solution of 2% *v*/*v* DMSO serving as the control. Eight batches of mosquitoes (i.e., 160 larvae in total) were exposed either to oregano oil or to carvacrol, and 4 batches (i.e., 80 larvae in total) were used in the control. The sum of moribund and dead larvae was considered for the mortality assessment 24 h after treatment, following WHO guidelines [45]. Live larvae 24 h after treatment were collected by means of a pipette, transferred using a wire gauze into plastic beakers with 100 mL distilled water, and fed ad libitum with a small piece of dried wheat bread until pupation. Water and food were replaced every day to avoid scum formation on the surface of the breeding medium. Every day, dead larvae and pupae were counted and removed from the beakers. Live pupae were collected daily and kept individually in plastic vials with water until adult emergence.

#### 2.4.2. Adult Longevity, Fecundity, Fertility, and Wing Length

Couples of 1-day old virgin males and females were placed in 1300 cm^3^ (length × width × height: 10 cm × 10 cm × 13 cm) plastic cages covered on the top with fine muslin, and were supplied with 10% sucrose solution through a cotton wick that was renewed once a week. The survival of coupled adults was recorded daily. The wing length of dead males and females was measured as body size index using a stereoscope, from the tip of the wing, excluding the fringe setae, to the bend in the trailing edge at the distal end of the alula [46]. A beaker of 100 mL water was provided as an oviposition substrate in the cage, while no blood meal was given to females. Pre-oviposition time was recorded, and records of the laid eggs (fecundity) and hatched larvae (fertility) were taken.

All bioassays were performed in room chambers at 26–27 °C, 50–60% relative humidity, and photoperiod (L:D) of 16:8 h.

#### 2.4.3. Data Analysis

In dose–response larvicidal bioassays, LC_50_ and LC_90_ values were determined through probit regression analysis [47]. The survival rates (%) of larvae and pupae that survived 24 h exposure of 3rd–4th instar larvae to LC_50_ concentrations of oregano oil and carvacrol were compared between the tested materials and control with the Pearson chi-square test. The survival rates (%) to adulthood of larvae that survived 24 h exposure to LC_50_ concentrations of oregano oil and carvacrol were compared between the tested materials and control with the Pearson chi-square test. The effects of the control and LC_50_ concentrations of οregano oil and carvacrol on larval, pupal, male, and female longevity, as well as on pre-oviposition period, laid eggs per female (fecundity), hatched larvae per female (fertility), and wing length of males and females, were analyzed with the non-parametric Kruskal–Wallis test (k samples), followed by pairwise comparisons in cases where significant differences were detected. The sex ratio of surviving adults (males:females), as well as the rates of fertile females, i.e., % of females that gave offspring, were compared between the treatments using the Pearson chi-square test [48]. All data analysis was performed at an α = 0.05 significance level with the statistical package IBM SPSS Statistics for Windows, version 21.0 (IBM Corp., Armonk, NY, USA).

## 3. Results

In the dose–response larva toxicity bioassays, oregano oil and carvacrol showed increasing mortality of 3rd–4th instar larvae of *Cx. pipiens* biotype *molestus* with increasing concentrations. Both tested materials exerted a high larvicidal effect, with LC_50_ values of 19.9 ppm (μL L^−1^) for oregano oil and 6.4 ppm (μL L^−1^) for carvacrol (Table 1).

**Figure 1:**
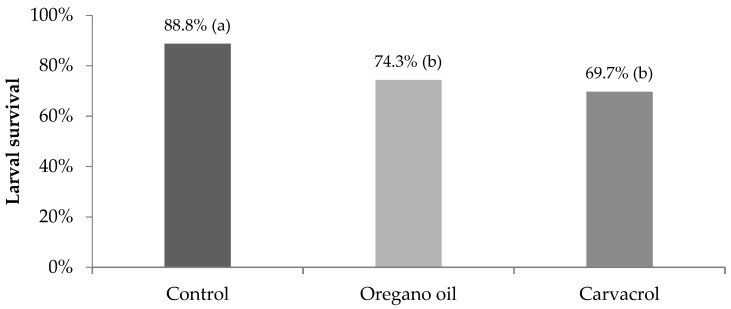
Larval survival (%) of *Cx. pipiens* biotype *molestus* mosquitoes that remained alive after 24 h exposure of 3rd–4th instar larvae to LC_50_ concentrations of oregano oil (*n* = 74) and carvacrol (*n* = 99), and to water solution of 2% DMSO (control) (*n* = 80). Percentages in a column followed by a different letter are significantly different (*p* < 0.05).

**Figure 2:**
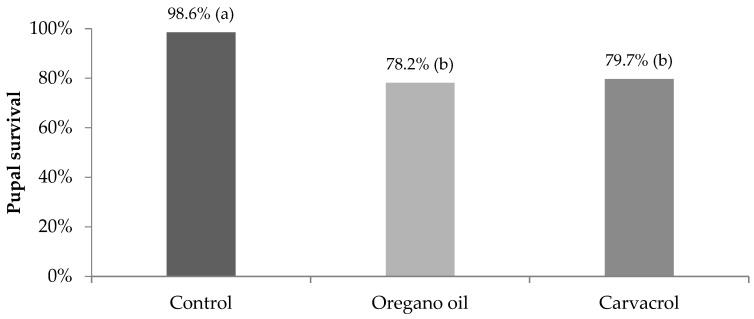
Pupal survival (%) of *Cx. pipiens* biotype *molestus* mosquitoes that remained alive after 24 h exposure of 3rd–4th instar larvae to LC_50_ concentrations of oregano oil (*n* = 55) and carvacrol (*n* = 69), and to water solution of 2% DMSO (control) (*n* = 71). Percentages in a column followed by a different letter are significantly different (*p* < 0.05).

**Figure 3:**
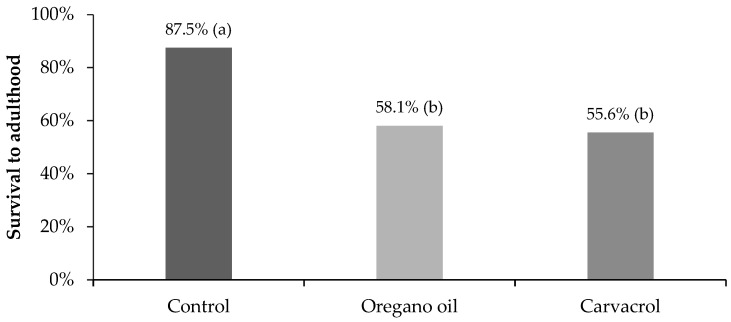
Survival (%) until adulthood of *Cx. pipiens* biotype *molestus* mosquitoes that remained alive after 24 h exposure of 3rd–4th instar larvae to LC_50_ concentrations of oregano oil (*n* = 74) and carvacrol (*n* = 99), and to water solution of 2% DMSO (control) (*n* = 80). Percentages in a column followed by a different letter are significantly different (*p* < 0.05).

After the application of the calculated LC_50_ concentrations of oregano oil (19.9 μL L^−1^) and carvacrol (6.4 μL L^−1^) on 3rd–4th instar larvae of *Cx. Pipiens* biotype *molestus*, 46.3% (74/160) and 61.9% (99/160) of the treated larvae survived after 24 h, respectively, while no mortality was observed in the control. The 24 h exposure of mosquito larvae to LC_50_ concentrations of both tested materials significantly reduced the percentage of surviving larvae that managed to pupate when compared to the control (Figure 1). Furthermore, the rate of surviving pupae that successfully turned into adults was significantly lower after oregano oil and carvacrol treatment in comparison to the control (Figure 2). Overall, only 58.1% and 55.6% of the larvae that survived after 24 h exposure to LC_50_ concentrations of oregano oil and carvacrol, respectively, finally reached adulthood, differing significantly from the survival rate to adulthood in the control (87.5%) (Figure 3). These findings indicate that the acute toxic larvicidal LC_50_ concentrations caused significant delayed mortality in surviving mosquitoes at the larval and pupal stages.

**Figure 4:**
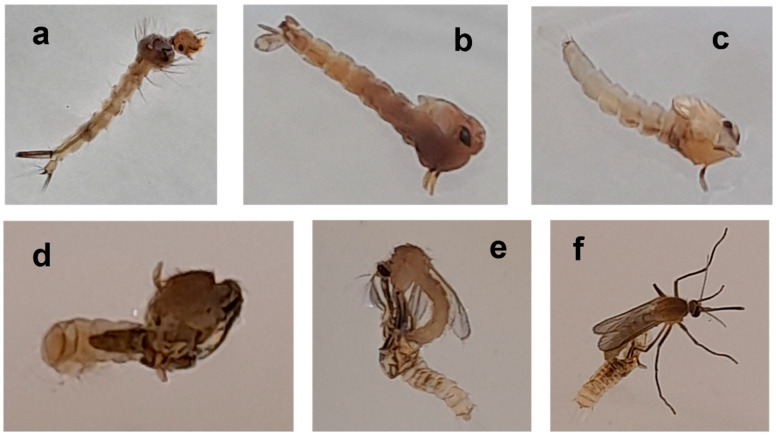
Dead elongated larva (**a**), larviform pupa (**b**), demelanized pupa (**c**), and failed adult emergence (**d**–**f**) after application of LC_50_ concentrations of oregano oil and carvacrol on 3rd–4th instar larvae of *Cx. pipiens* biotype *molestus*.

Interesting morphological abnormalities were observed in some cases after exposing larvae to LC_50_ concentrations of oregano oil or carvacrol: elongated dead larvae, larviform dead pupae being partially melanized or demelanized, and adults that failed to emerge properly and died on the water surface. Occasionally, only the head and thorax emerged from the puparium, or the adults were still attached to the puparium by one or more legs (Figure 4).

**Figure 5:**
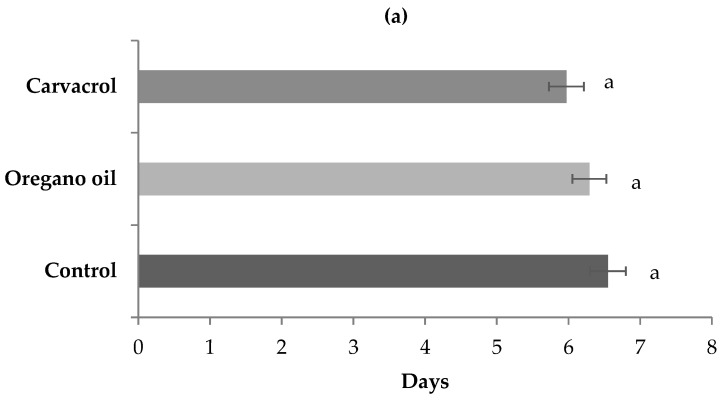

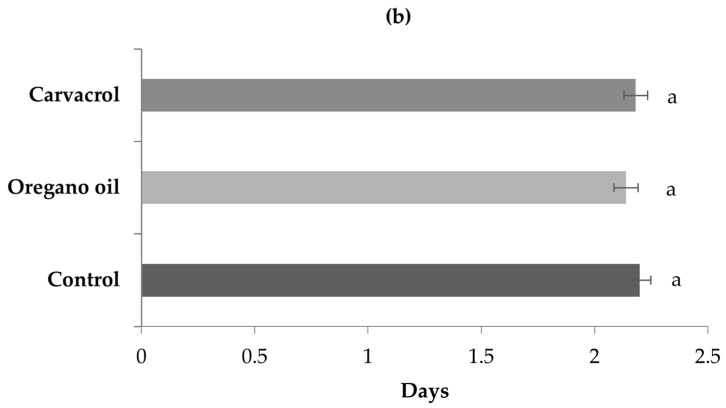
Mean (±S.E.M.) number of days for larval (**a**) and pupal (**b**) development of *Cx. pipiens* biotype *molestus* mosquitoes that remained alive after 24 h exposure of 3rd–4th instar larvae to LC_50_ concentrations of oregano oil and carvacrol, and to water solution of 2% DMSO (control). Means in a column followed by a different letter are significantly different (*p* < 0.05).

As shown in Figure 5, the treatment of 3rd–4th instar larvae with LC_50_ concentrations of both oregano oil and carvacrol for 24 h did not significantly affect the developmental time of the surviving larvae to pupation (*p* = 0.311) or, subsequently, the longevity of the surviving pupae until adulthood (*p* = 0.717), when compared to the untreated control.

The 24 h exposure of *Cx. pipiens* biotype *molestus* larvae to LC_50_ concentrations of oregano oil and carvacrol did not significantly affect the sex ratio of the surviving adults, females’ longevity, pre-oviposition period, fecundity, fertility, or wing length in both sexes. However, mosquito males that resulted from larvae treated with carvacrol lived for a significantly shorter time than the untreated ones (Table 2).

## 4. Discussion

Larvicidal dose–response laboratory testing showed promising results for carvacrol-rich oregano oil and pure carvacrol against *Cx. pipiens* biotype *molestus* larvae after 24 h exposure, as previously reported in toxicity bioassays with carvacrol and carvacrol-rich EOs from *Origanum vulgare* or other Lamiaceae plant species against the *Culex pipiens* mosquito species complex, including *Cx. pipiens* biotype *molestus* [28,29,30,31,32,33,34]. Acute and delayed mortality after the treatment of mosquito larvae with LC_50_ concentrations of the tested EO dominated by carvacrol (70% content) can be attributed to the action of the terpene, considering that the major components of essential oils at high concentrations (>20%) generally determine the biological properties of the essential oils [23,41].

The short-term (24 h) exposure of *Cx. pipiens* biotype *molestus* 3rd–4th instar larvae to LC_50_ concentrations of oregano oil and carvacrol caused significant delayed mortality in surviving larvae and pupae. These findings, along with observations of abnormal dead larvae and pupae and adults that failed to emerge, indicate the potential growth inhibitory activities of the tested EO and terpene interfering with the molting process. Phytochemicals extracted from several plant species show growth inhibitory effects on various developmental stages of different mosquito species, such as the prolongation of larval and pupal development, inhibition of larval and pupal molting, and delayed mortality, particularly during the molting and melanization process [49,50]. More commonly, as in our observations, the exposure of mosquitoes to phytochemicals may produce morphological abnormalities, such as demelanized cuticle at the larval and pupal stage, juveniles with elongated abdominal regions, dead larval–pupal intermediates with the head of a pupa and the abdomen of a larva (larviform pupae), and half-ecdysed adults unable to escape the pupal exoskeleton, which are signs of metamorphosis-inhibiting effects that are likely due to hormonal disturbance and/or interference in chitin synthesis during he molting process [50,51].

The results reported herein for *Cx. pipiens* biotype *molestus* align with our earlier findings, where the short-term (24 h) exposure of *Ae. albopictus* larvae to LC_50_ concentrations of the same oregano oil and carvacrol resulted in delayed toxicity and malformations at the larval and pupal stages, as well as adult emergence inhibition, suggesting insect growth regulatory (IGR) properties for the tested bioinsecticides [44]. The use of sublethal doses of oregano oil, dominated by d-pulegone, against 1st instar larvae of *Cx. pipiens* caused strong cumulative toxicity at the larval, pupal, and adult stages, significant prolongation of the larval and pupal periods, pupal and adult malformations, and strong inhibition of adult eclosion [52]. In contrast, the treatment of *Cx. pipiens* biotype *molestus* larvae with sublethal concentrations of oregano oil and carvacrol in the current study did not significantly affect the developmental time of the individuals that survived until pupation and adulthood. Similar to our study, growth inhibitory properties of plant secondary metabolites on different mosquito species have been reported in the past. *Citrus* EOs and their major component *R-(+)*-limonene exhibited IGR-like properties against *Ae. Albopictus*, considering that larvicidal sublethal concentrations caused a delayed killing effect and significant adult emergence inhibition [53]. Sublethal concentrations of *S-(−)*-limonene applied on the egg stage affected *Cx. pipiens* larval and pupal survival and development, significantly inhibiting adult emergence [54]. According to Dakhil and Morsy [55], the larvicidal action of LC_50_ doses of ethanolic lemon oil extract on *Cx. pipiens* extended from larvae to the resulting pupae, which were unable to escape from larval exuviae. The use of sublethal doses (LC_30_) of conifer EOs against *Cx. quinquefasciatus* 3rd instar larvae significantly reduced larval development and vitality to adulthood [56]. The exposure of *Anopheles* mosquito larvae to sublethal doses of emulsified *Ocimum kilimandscharicum* oil formulation, dominated by camphor and limonene, disrupted pupa formation, producing dead abnormal demelanized larval–pupal intermediates, prolonged the larval phase period, and caused failed adult emergence, thus suggesting insect growth regulatory bioactivities of the tested substance [57].

In the present study, the treatment of *Cx. pipiens* biotype *molestus* larvae with sublethal concentrations of oregano oil and carvacrol did not significantly affect the sex ratio of the surviving adults, female life span, fecundity, fertility, or wing length of adults. No significant sublethal effects of the tested materials on adult mosquito longevity, offspring production, or female wing length were reported against *Ae. albopictus* in our previous work either [44]. In contrast, the effects of some phytochemicals with growth regulatory action occasionally extend to the female progeny of exposed mosquito larvae by reducing the reproductive capacity, female survival, and body size [50,58]. The application of a sublethal LC_50_ dose of *Ipomea cairica* crude extract to 3rd instar larvae of *Ae. albopictus* and *Ae. Aedes aegypti* resulted in significantly lower egg production and hatchability in *Ae. albopictus* only, and reduced wing length of adults in both *Aedes* species [59]. The administration of LC_30_ concentrations of cinnamon EO, having (E)-cinnamaldehyde as its primary component, on *Cx. Culex quenquefasciatus* larvae for 12 h led to a significant reduction in the adult emergence and fertility of survived females [60]. Muthukrishnan and Pushpalatha [61] reported that *Cx. quinquefasciatus*, *Anopheles. stephensi* and *Ae. aegypti* larvae that survived from EI_50_ (adult emergence inhibition) concentrations of plant extracts from *Calophyllum inophyllum*, *Solanum suratense, Samadera indica*, and *Rhinocanthus nasutus* and managed to emerge as adults ultimately oviposited few eggs, most of which failed to hatch successfully. The exposure of *Ae. albopictus* and *Ae. aegypti* larvae to sublethal concentrations of crude extract of *Cyperus aromaticus* cultured cells increased the sterility and reduced the longevity and wing length of females [58]. When oregano EO rich in d-pulegone was applied at sublethal doses on larvae of *Cx. pipiens*, it drastically shortened adult longevity [52]. In our bioassays, mosquito males that were produced after larvicidal treatment with carvacrol lived for significantly less time than the untreated ones, thus suggesting a potential impact on mosquito population dynamics. However, male longevity was not affected by oregano oil treatment, likely due to the involvement of other components in the oil’s bioactivity.

Overall, the exposure of *Cx. pipiens* biotype *molestus* larvae to LC_50_ concentrations of carvacrol-rich oregano oil and pure carvacrol exerted an acute lethal effect on larvae 24 h after treatment, as well as significant delayed mortality for the larvae and pupae that survived until adulthood, affecting the longevity of the emerged males. Chronic growth inhibition effects along with observed abnormalities in larvae and pupae and failed adult emergence promote the insect growth regulatory potential of carvacrol against *Cx. pipiens* biotype *molestus*. Nevertheless, further biochemical and molecular studies are required to substantiate the insecticidal mode of carvacrol action. Our findings imply that the determination of the acute larvicidal effect after short-term (24 h) exposure of mosquito larvae may underestimate the overall efficacy of the tested substances. The considerable capacity of larvicidal botanicals in reducing mosquito adult emergence can be the critical endpoint potential against mosquito populations and transmission of vector-borne diseases such as WNV [50]. In this perspective, the effective doses of carvacrol and carvacrol-based EOs applied in water environments may be lower than the acute lethal concentrations, suggesting an eco-friendlier use against *Cx. pipiens* biotype *molestus*. Although EOs are generally considered eco-friendly substances, some may exert toxic negative effects on aquatic non-target organisms, such as the zooplankton *Dafnia magna*, depending on the applied doses and exposure time [62,63,64]. Reducing effective doses may be also of economic benefit for the mosquito control industry, considering the remarkable cost to produce plant secondary metabolites.

## Figures and Tables

**Table 1:** LC_50_ and LC_90_ values for the oregano oil and its major component carvacrol against 3rd to 4th instar larvae of *Cx. pipiens* biotype *molestus* in 24 h.

Tested Material	Slope (±SEM)	LC_50_ (95% CL) ^a^	LC_90_ (95% CL) ^a^	x^2^	d.f.
**Oregano oil**	5.11 ± 0.43	19.86 (16.97–23.48)	35.36 (28.60–53.81)	106.539 ^b^	22
**Carvacrol**	2.77 ± 0.30	6.41 (5.78–7.02)	18.57 (15.47–24.31)	21.165	26

^a^ LC values are expressed in μL L^−1^, and they are considered significantly different when 95% CL fail to overlap. ^b^ Since goodness-of-fit test is significant (*p* < 0.05), a heterogeneity factor is used in the calculation of confidence limits (CL).

**Table 2:** Effects on biological parameters of *Cx. pipiens* biotype *molestus* after 24 h exposure of 3rd–4th larvae to LC_50_ concentrations of oregano oil and carvacrol, and to water solution of 2% DMSO (control). Mean (±S.E.M.) longevity of males and females in days, pre-oviposition period in days, number of eggs per female (fecundity), number of larvae per female (fertility), and wing length of males and females in mm, as well as sex ratio of surviving adults (males:females) and % of fertile females (females that gave offspring).

Parameter	Control	Oregano Oil	Carvacrol	*p* Values
Sex ratio (males:females)	1.69:1 (70)	1.53:1 (43)	1.75:1 (55)	0.799
Male longevity (days)	25.8 ± 2.52 a (26)	23.6 ± 3.0 ab (16)	17.4 ± 2.53 b (19)	**0.041**
Female longevity (days)	32.9 ± 2.9 (26)	34.8 ± 2.5 (16)	29.8 ± 3.0 (19)	0.397
Pre-oviposition period (days)	5.7 ± 0.7 (23)	5.2 ± 0.5 (13)	4.9 ± 0.6 (15)	0.634
Fecundity (eggs per female)	45.4 ± 3.8 (26)	42.6 ± 6.6 (16)	32.3 ± 4.9 (19)	0.101
Fertility (larvae per female)	42.4 ± 4.4 (26)	37.8 ± 6.2 (16)	28.9 ± 5.1 (19)	0.092
Fertile females (%)	84.6 (26)	81.3 (16)	68.4 (19)	0.690
Wing length of males (mm)	2.67 ± 0.02 (23)	2.69 ± 0.013 (16)	2.68 ± 0.03 (17)	0.986
Wing length of females (mm)	3.20 ± 0.019 (25)	3.17 ± 0.022 (14)	3.18 ± 0.017 (19)	0.378

Values in bold represent significance at *p* < 0.05 level. Values in a row followed by different letters are significantly different (*p* < 0.05). Numbers in parentheses show the number of replicates.

## Data Availability

The data presented in this study are available upon request from the corresponding author.
